# Incontinentia pigmenti inherited from a father with a low level atypical *IKBKG* deletion mosaicism: a case report

**DOI:** 10.1186/s12887-022-03444-6

**Published:** 2022-06-29

**Authors:** Miki Kawai, Atsuya Sugimoto, Yasunori Ishihara, Takema Kato, Hiroki Kurahashi

**Affiliations:** 1 Division of Molecular Genetics, Institute for Comprehensive Medical Science, Fujita Health University, 1-98 Dengakugakubo, Kutsukake-cho, Toyoake, Aichi 470-1192 Japan; 2 Department of Clinical Genetics, National Hospital Organization Nagoya Medical Center, Nagoya, Aichi 460-0001 Japan; 3 Department of Neonatology, Japanese Red Cross Kyoto Daiichi Hospital, Kyoto, 605-0981 Japan; 4 Department of Pediatrics, Fukui Aiiku Hospital, Shinbo, Fukui, 910-0833 Japan

**Keywords:** Incontinentia pigmenti, *IKBKG*, Mosaicism, Paternal inheritance, Case report

## Abstract

**Background:**

Incontinentia pigmenti (IP) is an X-liked dominant genodermatosis caused by mutations of the *IKBKG*/*NEMO* gene. IP is mostly lethal in males in utero, and only very rare male cases with a somatic mosaic mutation or a 47,XXY karyotype have been reported.

**Case presentation:**

We here report a case of an *IKBKG* gene deletion in a female infant presenting with a few blisters and erythema in her upper arms at birth. MLPA analysis revealed a rare 94 kb deletion in this patient, encompassing the *IKBKG* gene and *IKBKGP* pseudogene. PCR analysis indicated the presence of *Alu* elements at both ends of the deletion, suggesting non-allelic homologous recombination as an underlying mechanism. Notably, a low-level mosaic deletion was identified in her father’s peripheral blood leukocytes by PCR, suggesting a rare father-to-daughter transmission of IP.

**Conclusion:**

In family studies for an apparently sporadic IP case, parental analysis that includes the father is recommended due to the possibility of male mosaicism.

## Background

Incontinentia pigmenti (IP; MIM #308300) is a rare disorder affecting 1.2 in 100,000 live births [1]. Skin lesions along Blaschko lines are observed in all IP patients and systemic involvement includes visual, dental and neurologic impairment. IP is an X-linked dominant disorder and is typically lethal in utero in males. However, rare affected males are occasionally live-born with severe ectodermal dysplasia and immunodeficiency (MIM#300291). Notably, very rare male cases with typical IP symptoms are similar to their female IP counterparts in that they are live-born harboring somatic mosaicism or with concomitant 47,XXY karyotype, Klinefelter syndrome [2, 3].

IP develops by a loss-of-function variant of the *IKBKG*/*NEMO* gene. The protein encoded by this gene, NEMO/IKKγ, is essential for the activation of the nuclear factor-kappa B (NF-κB) transcription factor. The cells lacking in the NEMO/IKKγ protein are sensitive to apoptosis, which contributes to lethality in males and selective skewed X-inactivation of the mutant allele in females [4, 5]. *IKBKG* is a 23-kb gene consisted of nine coding exons and four alternative non-coding first exons (1A-D), albeit with two promoters. In addition to the genuine *IKBKG* gene, a processed pseudogene, *IKBKGP*, encompasses the region between exons 3–10. The *IKBKGP* pseudogene constitutes a portion of a 35.7 kb segmental duplication that is located oppositely and next to each other. The most frequent pathogenic variant responsible for IP is a recurrent deletion generated by non-allelic homologous recombination (NAHR) due to an aberrant alignment between two 650 bp short interspersed nuclear elements, MER67B. This 11.7 kb deletion removes exons 4–10, thereby producing an early premature codon. The *IKBKG* region includes many repetitive sequences that are susceptible to rearrangement and other deletions have also been reported [6].

In our present case report, we describe an IP female with a novel *Alu*-mediated deletion inherited from a father with somatic mosaicism. We also discuss the clinical implications of this case for future genetic testing and counseling.

## Case presentation

### Clinical phenotype

The newborn female subject of this present study was the first child of a non-consanguineous healthy couple. Her fetal growth had stagnated throughout the late pregnancy period, and she was delivered after a 36-week and 4-day gestation period by cesarean section. She was 2079 g (− 1.3 SD) in weight, 43.5 cm (− 1.4 SD) in height, and had a 32.4 cm (±0 SD) head circumference and 29.0 cm chest circumference. Although her general body condition was good, she showed mild hypoglycemia of 26 mg/dL. A few blisters were observed inside her right upper arm and erythema was evident inside her left upper arm. The antimicrobial agent cefazolin was administrated due to a suspicion of infectious disease. Given her normal hepatic function, herpes virus was considered unlikely. After a few days however, the erythema and blisters had spread to the extremities.

Blood testing for inflammation, infection, and autoimmune disease was negative. A blood cultivation test further indicated no streptococcal, staphylococcal, or fungal infection of the nasal cavity, neck, ear, or affected skin. The use of antimicrobial agents was thus discontinued at 6 days old. The eruptions showed a decrease at 8 days old. Eosinophilia had increased since her birth from a few to more than 20%. At 11 days old, blister-like eruptions and erythema emerged again in a linear distribution on her lower limbs. IP was then strongly suspected based on this clinical progression. Secondary infections of the skin lesions were not present, and brain magnetic resonance imaging was normal. The polus posterior lentis on both eyes showed mild whitening but did not require surgical treatment. The paternal grandmother subsequently remembered that the father of our infant subject had also had minor skin lesions at birth.

### Molecular analysis

**Fig. 1:**
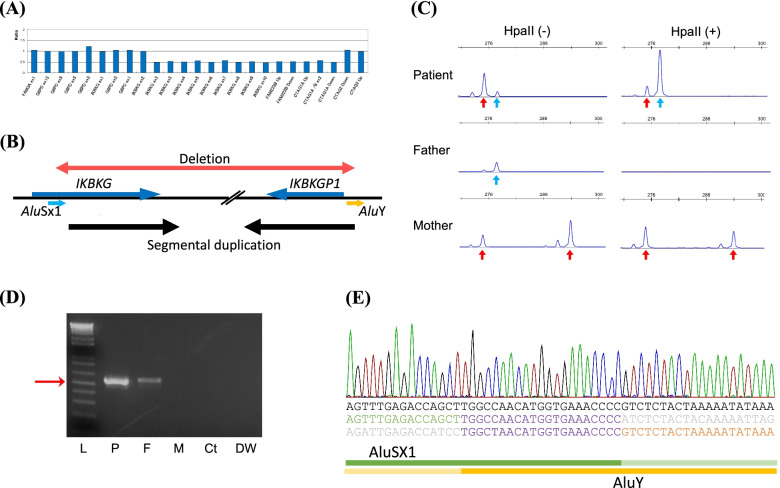
Molecular analysis of the father-to-daughter inheritance of IP in the study case. A Diagram of the quantitative MLPA results. Genomic regions are displayed on the x-axis, whereas the y-axis indicates the ratio to a normal female. The genomic positions of the probes are provided in the instruction manual of the MLPA kit (SALSA P073-A1, MRC-Holland). **B** Schematic depiction of the location of the deletion relative to the *IKBKG* gene and *IKBKGP* pseudogene. The red arrow indicates the deleted region in the study patient. The cyan arrow indicates *Alu*Sx1, and the yellow arrow denotes *Alu*Y. Black arrowheads indicate segmental duplications. *Alu*Sx1, GRCh38/hg38 chrX:154554478–154,554,769; *Alu*Y, 154,648,589–154,648,883; *IKBKG*: 154547620–154,565,033; *IKBKGP1*: 154639978–154,648,275; deletion: 154554588–154,648,697. **C** Electropherogram of the HUMARA assay. *Hpa*II undigested (left) and digested (right) PCR products from the patient and her parents are shown. (Above) The red arrowhead indicates the maternal allele, and the cyan arrowhead denotes the paternal allele. (Middle) The father’s X chromosome allele. (Bottom) The mother’s two alleles. **D** Deletion-specific PCR. P, proband; M, mother of the affected proband; F, father of the affected proband; DW, distilled water; L, 1 kb ladder plus (Invitrogen). The red arrowhead indicates a nested PCR product. **E** Deletion junction sequence. The green and orange characters indicate the upstream and downstream *Alu* elements, respectively. The purple character sequences indicate an identical 16 nucleotides. Gray character sequences indicate the region deleted in the variant

To further diagnose our current case subject, we performed PCR analysis of the 11.4 kb recurrent deletion in the *IKBKG* gene that is commonly observed in IP patients [7]. No specific PCR product was obtained in the proband however (data not shown). We next performed sequencing analysis of all coding exons of the *IKBKG* gene using the Sanger method, but no pathogenic variant was identified (data not shown). We subsequently conducted multiplex ligation-dependent probe amplification (MLPA) (P073-A1; MRC-Holland, Amsterdam, Netherlands) to detect possible rare genomic rearrangements, and identified a large deletion incorporating exons 3–10 of the *IKBKG* gene, as well as a similar region of the *IKBKGP* pseudogene, based on a copy number evaluation of the MLPA data (Fig. 1A, B). Neither parent showed a copy number abnormality in this test.

The results of a human androgen receptor assay (HUMARA) indicated a skewed X chromosome inactivation in the proband (Fig. 1C) [8]. This inactivated allele was inherited from the father, suggesting either that the proband’s deletion arose on the paternal allele as a de novo event or that the father may also harbor a mosaic deletion that is difficult to detect using MLPA. To examine for the presence of a possible low level mosaic deletion in the father, we established a deletion-specific PCR assay, which is more sensitive than MLPA, by designing the appropriate primers (Fig. 1B, Table 1). We tried some sets of forward and reverse primers located outside the deleted exons, not on the segmental duplication. This PCR approach successfully amplified a 3 kb product in the proband. Notably, a smaller but significant amount of this same PCR amplicon was observed in whole blood leukocyte DNA obtained from her father. Nested PCR was thus performed and confirmed a low-level mosaic deletion in the father (Fig. 1D). Analysis of the paternal blood DNA by quantitative PCR indicated that the mosaic deletion ratio was as low as approximately 3.3%. The Sanger sequence of the PCR product showed that both deletion endpoints were located within the *Alu* repeat elements. The proximal endpoint belonged to *Alu*Sx1 (292 bp), and the distal endpoint to *Alu*Y (295 bp), which share an 83.6% sequence similarity. The putative breakpoints were near the center of these elements and an identical 16 nucleotides were observed at the junction (Fig. 1E). These results indicated that the genomic position of the 94 kb deletion was GRCh38/hg38 chrX:154554588–154,648,697.

**Table 1:** PCR primers used for the *IKBKG* deletion-specific PCR

		Nucleotide
First PCR	Forward primer	AGGACAGCCTTGCTGGATCTTAC
	Reverse primer	GTCTTCCTTCCTGAAGCTGTGTCT
Nested PCR	Forward primer	GAGGCAGATGCAGATGAGGAGG
	Reverse primer	CTACCGAGGCAAGAAGATCGCT
Quantitative PCR	Probe	TTCATGCCATTCTCCTGCCTCAGCCTCC
	Forward primer	TGAGGGAGAGATTCACGAAGAAGT
	Reverse primer	CTGCGGCCATCTGTTTTTGC

## Discussion and conclusions

We here describe a rare IP case involving a father to daughter inheritance of a novel pathogenic *IKBKG* deletion. Our analysis indicated that the paternal pathogenic variant was a low-level somatic mosaicism, which was why the child’s father did not suffer lethality at the fetal stage of development. Such a father-to-daughter transmission is very rare with only two such cases reported to date [9]. In both of these prior cases, the female probands manifested typical IP symptoms and the father of each child also had mild IP skin symptoms. DNA testing of the peripheral blood as well as of fibroblasts from each father of those previous patients showed no evidence of an *IKBKG* mutation, but the sperm DNA carried the proband’s mutation with a 16.7 and 35% germline mosaicism, respectively. This indicated that the mutation arose before differentiation of the primordial germ cells and that the mutant germ cells survived while many mutant cell populations declined in the other somatic tissues under negative selection pressure. In this context, the fact that the father in our current case carried a 3.3% level of mosaicism in his blood may suggest more mutations in his sperm.

In our present case, the father presented with a typical cutaneous rash at birth, but since these skin lesions disappeared with age, he did not realize that he had had IP symptoms until the birth of his daughter. Given that a mosaic mutation can be inherited by offspring, diagnosing an IP male with a mild skin manifestation may be difficult but is important. Even if a proband seems to be a sporadic case without any family history, parental analysis that includes the father is recommended due to the possibility of paternal mosaicism. In terms of genetic counseling in these cases, pediatricians or genetic specialists need to inform the apparently healthy parents the possibility of hidden mosaicism and the recurrence risk of IP in next pregnancy, and also consider how to inform the affected child of future reproductive issues when he or she reaches adulthood.

Besides the typical recurrent deletion of the *IKBKG* gene, various genomic rearrangements in the *IKBKG* region, both benign and pathogenic, have also been reported [6]. A total of 7 pathogenic deletions ranging from 4.8–115 kb in size have been described. The breakpoint locations of these cases were different from that of our case. In our present analyses, deletion junctions were analyzed to predict a mechanism from a wide variety of possibilities, including non-homologous end joining, NAHR mediated by *Alu*, and replication-based processes. Since both endpoints in our present female infant subject were located within 300 bp *Alu* elements with a 83.6% sequence similarity, *Alu*-mediated NAHR may have been the underlying mechanism in this case. Among the 7 atypical deletions reported previously, the precise breakpoint was identified only in one case, whose deletion was found to be mediated by *Alu*-*Alu* recombination [6]. In contrast, common recurrent exons 3–10 deletions are caused by recombination between the two MER67B elements located in intron 3 and downstream to exon 10 of the *IKBKG* gene, respectively, and the endpoints are located within 879 bp of MER67B-surrounding regions that have a 100% sequence identity. The 100% identity might explain the difference in the de novo frequencies of these deletions.

## Data Availability

The data of mutation identified during the current study are available in the Leiden Open Variation Database repository, Variant ID: #0000868293.

## Abbreviations

IP: Incontinentia pigmenti
*IKBKG*: inhibitor of kappa polypeptide gene enhancer in B-cells, kinase gamma; *NEMO* nuclear Factor κB, essential modulator
NF-κB: nuclear factor-kappa B
*IKBKGP*: inhibitor of kappa polypeptide gene enhancer in B-cells, kinase gamma pseudogene
NAHR: non-allelic homologous recombination
MER67B: medium reiterated 67B
MLPA: multiplex ligation-dependent probe amplification
HUMARA: human androgen receptor assay
